# Microstructure and Mechanical Properties of AlSi7Mg0.6 Aluminum Alloy Fabricated by Wire and Arc Additive Manufacturing Based on Cold Metal Transfer (WAAM-CMT)

**DOI:** 10.3390/ma12162525

**Published:** 2019-08-08

**Authors:** Qingfeng Yang, Cunjuan Xia, Yaqi Deng, Xianfeng Li, Haowei Wang

**Affiliations:** 1 School of Materials Science and Engineering, Shanghai Jiao Tong University, 800 Dongchuan, Shanghai 200240, China; 2 The State Key Laboratory of Metal Matrix Composites, Shanghai Jiao Tong University, Shanghai 200240, China

**Keywords:** wire and arc additive manufacturing, cold metal transfer, AlSi7Mg0.6, microstructure, mechanical properties

## Abstract

Wire and arc additive manufacturing based on cold metal transfer (WAAM-CMT) has aroused wide public concern in recent years as one of the most advanced technologies for manufacturing components with complex geometries. However, the microstructure and mechanical properties of the parts fabricated by WAAM-CMT technology mostly are intolerable for engineering application and should be improved necessarily. In this study, heat treatment was proposed to optimize the microstructure and enhance mechanical properties in the case of AlSi7Mg0.6 alloy. After heat treatment, the division between coarse grain zone and fine grain zone of as-deposited samples seemed to disappear and the distribution of Si and Mg elements was more uniform. What is more, the yield strength and ultimate tensile strength were improved significantly, while the ductility could be sustained after heat treatment. The improvement of strength is attributed to precipitation strengthening, and the shape change of Si phase. No reduction in ductility is due to the higher work hardening rate caused by nanostructured precipitate. It is proved that heat treatment as an effective method can control the microstructure and enhance comprehensive mechanical properties, which will boost rapid development of WAAM industrial technology.

## 1. Introduction

Due to the excellent characteristics of low density, high strength, good thermal conductivity and corrosion resistance, aluminium alloys have been widely used in various applications, among which the amount of Al-Si alloy (especially Al-Si-Mg alloy) accounts for more than half. Al-Si alloy, an ideal casting alloy material, has good liquidity, castability, corrosion resistance, and low tendency to produce casting crack, which has become one of the most popular structural materials in the manufacturing industry [1,2,3,4,5,6]. 

Additive manufacturing (AM), which manufactures the components through the deposition of materials layer-by-layer, instead of conventional material removal methods, has gained worldwide popularity over the past thirty years [7]. This technology can reduce production processes and avoid materials waste observably, meeting the development direction of energy saving and high efficiency [8,9]. Besides, it enables rapid prototyping of any complex shape theoretically [10]. According to the heat source, additive manufacturing techniques can be classified as laser additive manufacturing (such as selective laser melting (SLM) [11]), arc additive manufacturing (such as gas metal arc-additive manufacturing [12]), and electron beam additive manufacturing (such as electron beam rapid manufacturing (EBRM) [13]). In addition, additive manufacturing technologies can be classified as either a powder-feed process or a wire-feed process when it comes to how the additive material is supplied [14,15]. Though the powder-feed process is capable of manufacturing products with high geometrical accuracy and complex shape, it has disadvantages of low deposition rate (0.1–0.2 kg/h) low material utilization and high cost. While the wire-feed process has higher deposition rate (5 kg/h) lower cost and higher material utilization compared with the powder-feed process [16,17]. What is more, the wire-feed process is a cleaner and more environmentally friendly process in spite of its low forming precision, which makes itself have competitive advantages and prospect. Among these additive manufacturing techniques, wire and arc additive manufacturing (WAAM) is becoming more and more popular due to its low production cost, high deposition rate and the capability of fabricating large-scale components [18,19]. Wire and arc additive manufacturing based on cold metal transfer (WAAM-CMT) technology has the advantages of small heat input, small deformation, high welding speed and low operation cost. Because of these advantages, it is very suitable for the research of low melting point metal (such as aluminium alloy) [20,21]. 

However, most of the time rapid cooling microstructure (such as oriented grains and elements segregation, etc.) of metallic materials fabricated by AM technology is detrimental to service performance. For example, E. Chlebus et al. [22] found that there was the segregation of Nb, Mo elements and texture in the microstructure of Inconel 718 alloy fabricated by selective laser melting. Distinct composition segregation was also found by Bai et al. [23] in 4043 aluminum alloy fabricated by wire and arc additive manufacturing (WAAM). Besides, Zhang et al. [24] found large columnar crystals grew along interlamellar orientation and there was obvious anisotropy in the samples. On the other hand, residual stress is likely to exist in the samples because of the non-uniform temperature field during the process of additive manufacturing [25], so the performance of the components fabricated by additive manufacturing is often unsatisfactory. In recent years, it is found that heat treatment as one of the effective means could enhance the microstructure and weaken anisotropy of metallic materials fabricated by AM. For instance, R. Wauthle et al. [26] studied the effects of heat treatment on the microstructure and mechanical properties of Ti-6Al-4V fabricated by SLM, founding hot isostatic pressing (HIP) could transform tissues into isotropic lamellar α + β phases and improve plasticity effectively. E. Chlebus et al. [22] fabricated Inconel 718 Ni-based alloy using SLM and found that solid solution could homogenize the γ austenite matrix and improve the mechanical properties significantly. The advantage of heat treatment lies in that heat treatment does not affect the process of additive manufacturing. Besides, people have a large number of known test data of various metal materials for reference, because heat treatment is a kind of material modification method with a long history. However, it is not clear whether the existing heat treatments can enhance the structure of the sample fabricated by AM effectively or not. There is still the necessity to explore new heat treatment methods for enhancing the mechanical properties of additive manufacturing materials [27,28,29,30,31]. 

Therefore, it is of great significance to study the effect of heat treatment on metal materials made by additive manufacturing for the extensive application of additive manufacturing technology. However, most of the researches focus on SLM technology about additive manufacturing Al-Si alloy at present and there are few researches on Al-Si alloy fabricated by cold metal transfer (CMT). Some researches focus more on process parameters of CMT technique and pay little attention to microstructure and mechanical properties of materials processed by CMT, let alone the potential physical mechanisms of controlling microstructure architecture and improving mechanical properties under the effect of heat treatment. Therefore, in this study, AlSi7Mg0.6 aluminium alloy as a model alloy is used to explore the effects of heat treatment on microstructure and mechanical properties using WAAM-CMT technology. The possible physical mechanisms of optimizing microstructure and enhancing mechanical properties are discussed in detail.

## 2. Materials and Methods

**Figure 1:**
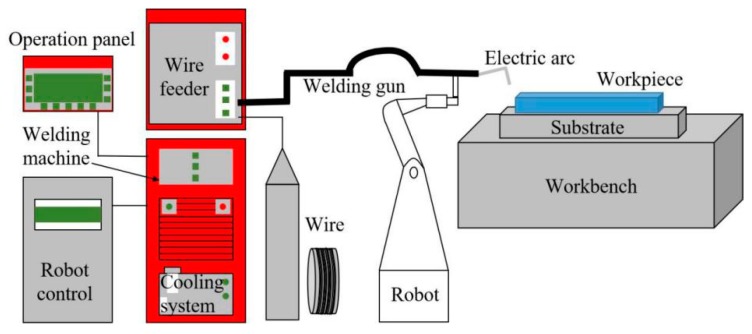
The sketch map of the WAAM-CMT equipment.

**Figure 2:**
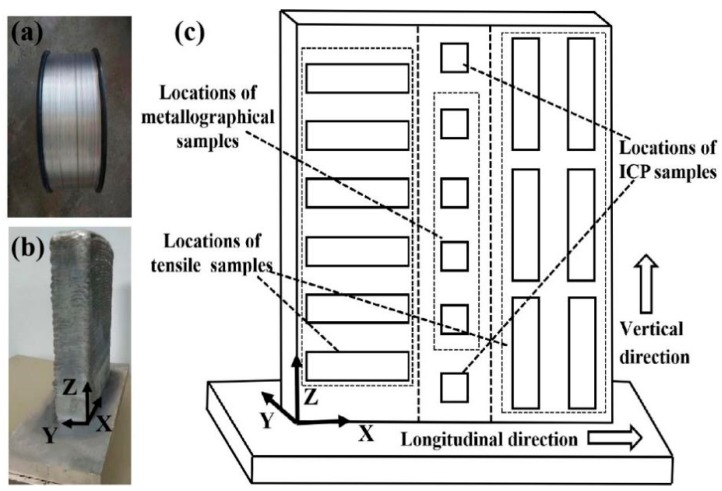
(**a**) The wire used in wire and arc additive manufacturing based on cold metal (WAAM-CMT); (**b**) The samples fabricated by WAAM-CMT; (**c**) Specimens extraction locations and definition of three directions (X, Y, Z).

In this study, the rapid prototyping system was mainly composed of Metal-Inert Gas (MIG) welding machine, welding robot and robot control cabinet. The sketch map of the equipment is shown in Figure 1. In the process of rapid prototyping, 4043 aluminum alloy with a size of 290 × 120 × 20 mm^3^ was used as the base plate, the diameter of the AlSi7Mg0.6 wire was 1.6 mm (Figure 2a), and pure argon was used in the whole process. Before the test, the aluminum alloy substrate was cleaned with alkaline solution to remove oil stains firstly, followed by mechanical polishing to remove the surface oxidation film, and wiped clean with acetone finally. 

Welding current of 92 A, welding voltage of 11.8 V, travel speed of 9 mm/s and wire feed speed of 5.7 m/min were used to fabricate the thin-wall sample with dimensions 140 × 35 × 172 mm^3^ in X, Y, and Z directions, respectively (Figure 2b). Three directions are defined by this way: X-along the direction of the travel of the welding torch, Y-perpendicular to the direction of the travel of the welding torch, and Z-along the build direction. Specimens were extracted from the location as shown in Figure 2c. In addition, the chemical composition of the wire and the samples is shown in Table 1. T6 heat treatment (solid solution 535 °C/1 h + quenching + artificial aging 120 °C/1 h + 165 °C/8 h) were carried out on the printed samples, and the quenching method was water-cooling. 

In order to prepare specimens for metallographic analysis, the as-deposited specimens were firstly ground and polished with sandpaper. After that, the samples were cleaned with alcohol in the ultrasonic cleaner for 10 min and finally corroded with Keller reagent (1 vol.% HF + 1.5 vol.% HCl + 2.5 vol.% HNO_3_ + 95 vol.% H_2_O) for 15 s. The optical microscopy (OM, AXIO Imager A2, Zeiss, Oberkochen, Germany) and scanning electron microscope (SEM, Tescan, FERA3 XMU/XMH, Brno, Czech Republic) were used for microstructure characterization. What’s more, electron backscattered diffraction (EBSD, Tescan, FERA3 XMU/XMH, Brno, Czech Republic) technique was used for statistical analysis of grain size as well as the grain orientation analysis and energy disperse spectroscopy (EDS, Tescan, FERA3 XMU/XMH, Brno, Czech Republic) was used for elements analysis. The EBSD samples were prepare by three ion beam cutting technique (Leica EM TIC 3X, Wetzlar, Germany). For composition analysis, iCAP6300 (Thermo Fisher Scientific, MA, USA) inductively coupled plasma optical was used. Finally, tensile tests were carried out in universal testing machine (Zwick/RoellZ100, Ulm, Germany) at ambient temperature with the strain rate 1 × 10^−4^ s^−1^. 

## 3. Results

### 3.1. Microstructure Evolution

**Figure 3:**
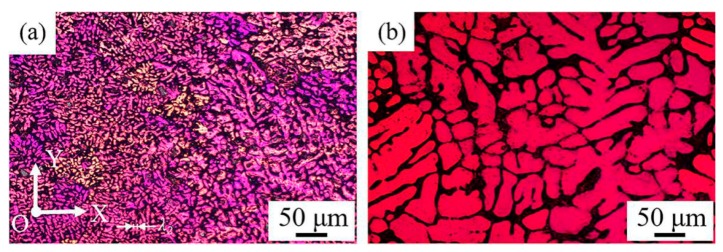
(**a**) Grain structure in face XOY of the as-deposited samples; (**b**) Grain structure of casting AlSi7Mg0.6 alloy.

Figure 3a shows the grain structure in face XOY of the as-deposited samples. It can be seen clearly that the grain size and the dendrite arm spacing in the as-deposited samples are obviously finer than those of the ordinary casting samples (Figure 3b). This may be caused by the different cooling rates in two processing technologies. The cooling rate during solidification can be determined with the secondary dendrite arm spacing or mean length of cell boundaries. The relationship between the secondary dendrite arm spacing or the length of cellular grain and the cooling rate can be expressed with the empirical formulate as follows:

(1) $$\lambda_{2}=B\times R^{-n}$$

where $\lambda_{2}$ is the length of cellular grain or secondary dendrite arm spacing (m), *R* is the cooling rate (Ks^−1^), and *B* and *n* are constants which are determined by material and process. For Al alloys, *B* and *n* are typically about 5 × 10^−5^ (m (Ks^−1^)^1/3^) and 1/3, respectively [32,33]. The $\lambda_{2}$ is determined by taking an average value of the length of cellular grain or secondary dendrite arm spacing from the deposited layer. According to Figure 3a, the secondary dendrite arm spacing of the samples is about 8 μm, and the cooling rate of the WAAM-CMT process is about 244 K/s, which is much higher than the cooling rate of ordinary casting process.

**Figure 4:**
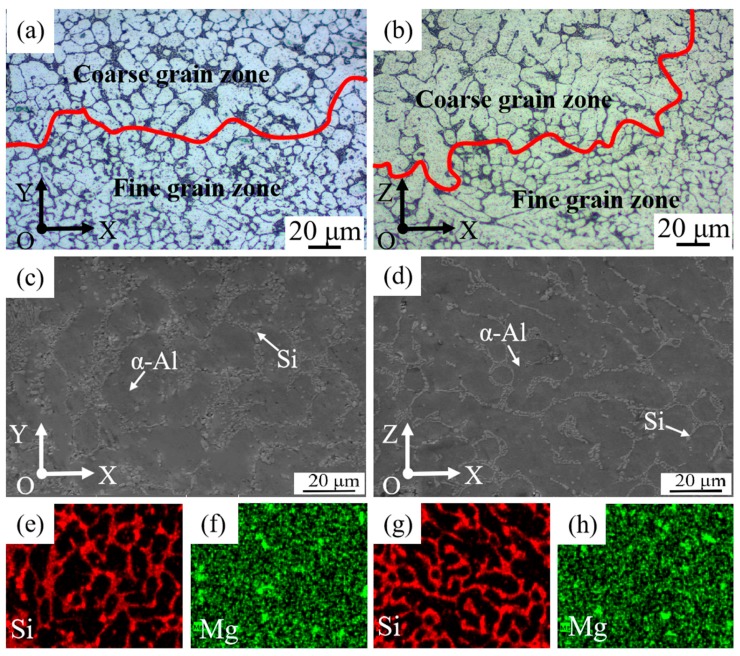
Metallographic morphology, scanning electron microscope (SEM) and energy disperse spectroscopy (EDS) graphs of the as-deposited samples: (**a**) face XOY; (**b**) face XOZ; (**c**) face XOY; (**d**) face XOZ; (**e**,**f**) Distribution of Si, Mg elements of (**c**) respectively; (**g**,**h**) Distribution of Si, Mg elements of (**d**) respectively.

Though fine dendrite arm spacing can be obtained by WAAM-CMT technology, the microstructure of the as-deposited samples is inhomogeneous, as can be seen in Figure 4. Figure 4a,b show the metallographic morphology in face XOY and face XOZ of the as-deposited samples. The microstructure in two faces of the as-deposited samples is similar, consisting of columnar and equiaxed grains. Besides, there is a clear division between coarse grain zone and fine grain zone and the columnar grains in face XOZ are more obvious than that in face XOY. In addition, α-Al phase is mainly dendritic and Si phase is mainly distributed along grain boundaries with a needle-like or rod-like shape. Figure 4c–h show the microstructure and the elements distribution in face XOY and face XOZ of the as-deposited samples and it can be seen that there is obvious elements segregation in the as-deposited samples. Si phase is distributed along the grain boundary, which means that Si mainly exists in the form of eutectic Si. 

**Figure 5:**
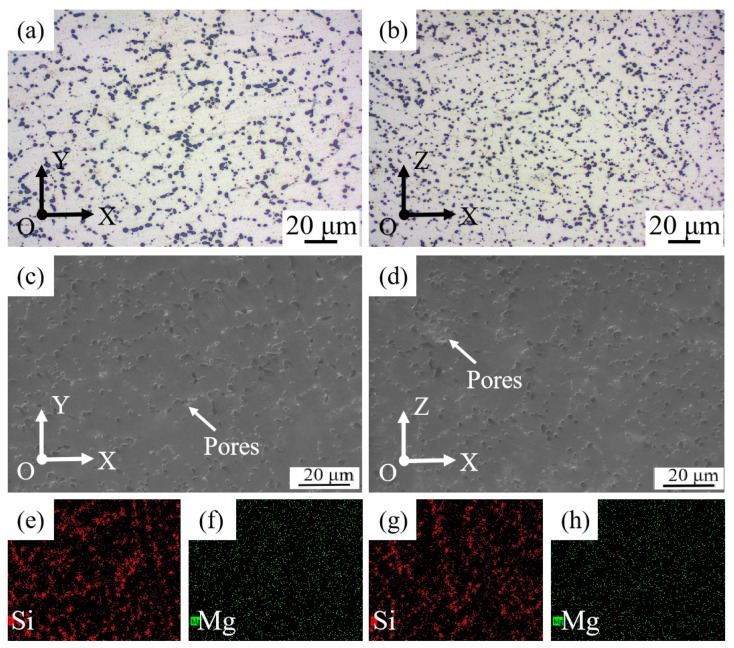
Metallographic morphology, SEM and EDS graphs of the T6 heat-treated samples: (**a**) face XOY; (**b**) face XOZ; (**c**) face XOY; (**d**) face XOZ; (**e**,**f**) Distribution of Si, Mg elements of (**c**) respectively; (**g**,**h**)Distribution of Si, Mg elements of (**d**) respectively.

After T6 heat treatment, though there is difference in the distribution of Si, the inhomogeneity of microstructures of the as-deposited samples has been improved greatly (Figure 5a,b). The dendrite morphology of α-Al phase becomes less obvious, and the grain size increases. What’s more, the division between coarse grain zone and fine grain zone seems to disappear after T6 heat treatment. The eutectic silicon phase becomes rounded obviously, and Si is distributed along the grain boundary discontinuously with a globular or bulky shape, as shown in Figure 5c,d. The size of Si phase is more uniform, about 3–7 μm. Besides, the phenomenon of large-scale segregation of elements in grain boundary has disappeared, the distribution of Si and Mg elements is more uniform (Figure 5e–h), especially for Mg element. In addition, there are pores in the samples, and the size of the pores is about 2–4 μm, which may be caused by the gas existed in the wires or absorbed from the atmosphere during the manufacturing process. 

**Figure 6:**
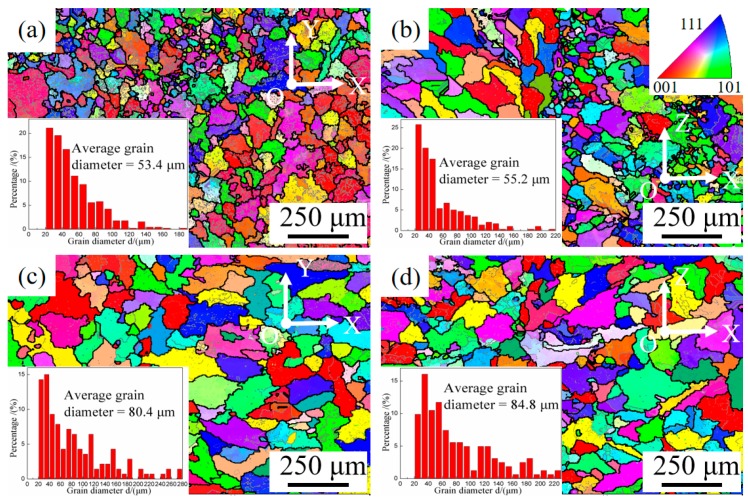
Electron backscattered diffraction (EBSD) graphs and statistical diagrams of grain size distribution of samples in different states: (**a**) face XOY of the as-deposited samples; (**b**) face XOZ of the as-deposited samples; (**c**) face XOY of the T6 heat-treated samples; (**d**) face XOZ of the T6 heat-treated samples.

Figure 6a,b show that although the average grain size in the two faces is very similar, there is a certain difference in the grain distribution in face XOY and face XOZ of the as-deposited samples. The grain size distribution is more even in face XOY and most of the grains in face XOY are equiaxed with smaller size. While in face XOZ, there are more columnar grains with larger grain size, which may be related to the WAAM-CMT process and the reason will be analyzed in the discussion section. Figure 6c,d show that the grain sizes in face XOY and face XOZ of the T6 heat-treated samples both increase obviously compared with that of the as-deposited samples, an increase of 50.56% and 53.62% respectively. Furthermore, the obvious division line of coarse grains and fine grains is almost invisible.

### 3.2. Mechanical Properties Analysis

**Figure 7:**
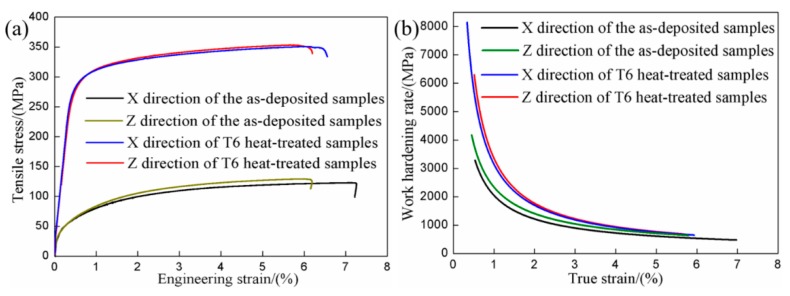
(**a**) Tensile test of the samples in X and Z directions fore-and-aft heat treatment; (**b**)Work hardening rate plotted vs. true strain of the samples in X and Z directions fore-and-aft heat treatment.

The tensile test results and the mechanical properties of the samples are summarized in Figure 7 and Table 2. Tensile force was applied along X and Z directions in order to investigate the influence of loading orientation on the tensile properties. The tensile test results have shown that there is basically no difference in the yield strength and ultimate tensile strength in X and Z directions of the as-deposited samples considering the existence of experimental errors and there is a certain difference in the elongation in two directions. After T6 heat treatment, the ultimate tensile strength and elongation of the samples are basically the same in X and Z directions, though there is a difference in the yield strength in two directions.

After T6 heat treatment, the elasticity modulus (m_E_), the yield strength (R_p0.2_) and the ultimate tensile strength (R_m_) of the samples in X direction has an increase of 37.79%, 272.46% and 181.22% respectively, and those in Z direction increases 41.97%, 318.37% and 171.41%, respectively. The elongation (A) of the samples in X direction has a decrease by 7.49%, while that in Z direction is basically unchanged. It indicates that the mechanical properties of AlSi7Mg0.6 aluminum alloy produced by WAAM-CMT have been improved obviously by T6 heat treatment.

In order to further quantify the work hardening response, Figure 7b shows the work hardening rate plotted vs. true strain of the samples in X and Z directions before and after heat treatment. Work hardening rate is calculated by the following formula [34]:

(2) $$\Theta =\frac{\partial \sigma }{\partial \varepsilon }$$

where Θ is the work hardening rate (MPa), *σ* is true stress during the uniform plastic deformation stage (MPa), *ε* is true strain correspondingly (%). *σ* and *ε* during the uniform plastic deformation stage in tensile curve is fitted by the equation [35,36]:

(3) $$\sigma =K\times \varepsilon^{n}$$

where *n* is the work hardening coefficient and *K* (MPa) is the strength coefficient, which represents the increment in strength due to work hardening with *ε* = 1.

It can be seen that no matter what state the sample is in, work hardening rate curves can be divided into three stages. When true strain is less than 1.5%, work hardening rate of the samples decreases sharply as the true strain increases. When true strain is greater than 1.5% and less than 4%, the decrease rate of work hardening rate declines with the increase of true strain. When true strain is greater than 5%, work hardening rate of the samples tends to be a stable value. Although work hardening rates of as-deposited and T6-heat treated samples in two directions all decrease with increasing tensile strain, work hardening rate of T6-heat treated samples is higher than that of the as-deposited samples. Besides, there is a certain difference between the work hardening rate curves in X and Z directions of the as-deposited samples, and the work hardening rate curves in two directions of the T6 heat-treated samples is almost overlapping.

## 4. Discussion

### 4.1. Analysis of the Microstructure 

The refining effect of aluminum alloy mainly manifests in the refinement of the α-Al phase, such as the refinement of grain size and the reduction of secondary dendrite arm spacing [37]. The dendrite arm spacing of the as-deposited samples is obviously finer than that of the ordinary cast samples, which is determined by the process of WAAM-CMT. For one thing, the cooling rate of WAAM-CMT process is high, which can form fine grain. For another, the agitating effect of arc on molten pool also promotes grain refinement. Some specialists claim that the grain size of samples produced by the CMT arc mode has finer size than that produced by other arc mode. For instance, Wang et al. [38] claimed that high arc pressure induced by pulse arc mode could produce enough oscillation to break the dendrite arms, providing more heterogeneous nucleation sites and refining the grain size. Li et al. [39] indicated that the electromagnetic force of CMT mode was larger than other mode, resulting in stronger stirring effect in the melt pool and smaller grain size. 

Though fine dendrite arm spacing obtained by WAAM-CMT technology is beneficial to the mechanical properties of samples, there is a clear division between coarse and fine grains and a mixture of columnar crystal and isometric crystal of the as-deposited samples, which is harmful to the mechanical properties of samples. This is related to the solidification process. Since the bottom of the molten pool is in contact with the deposited weld bead, the heat flows from the deposited weld bead to the substrate, which is the direction of the maximum temperature gradient. Therefore, the growth direction of the dendrites is opposite to the direction of heat flow, forming columnar dendrites. Due to the fast cooling rate of molten pool and the large temperature gradient, the columnar dendrites grow rapidly at the bottom of the molten pool and the grain size is relatively large. However, the temperature gradient in the middle of the molten pool decreases gradually and the crystallization rate increases gradually, forming smaller dendrites. The top of the molten pool is in contact with air and the temperature gradient is small, forming equiaxial crystal. Besides, when the molten pool is forming at the upper layer, the top zone of the deposited weld bead will melt again. Therefore, the microstructure of the as-deposited samples is a mixture of coarse and fine crystals, columnar dendrites and equiaxial crystal eventually [40,41]. The above is also the reason why columnar grains existing in face XOZ has a large proportion. When the arc moves along X direction, there is also a higher temperature gradient along the direction of the newly solidified area at the same layer, resulting in more even grain size in face XOY. Besides, the elements segregation is also caused by the high cooling rate of WAAM-CMT process. When cooling rate of solidification is high, the elements are not sufficiently dispersed, resulting in elements segregation in the final solidification position [23,41].

After T6 heat treatment, the average grain sizes in face XOY and face XOZ both increase obviously. The growth of grains occurs in a form of the migration of large angle grain boundary. In the process of grain growth, larger grains usually devour smaller grains. The driving force of grain growth is the decrease of the total free energy of the system after grain boundary migration [42]. 

Additionally, the changes of the morphology of Si phase and distribution of elements after T6 heat treatment are caused by the diffusion of elements. According to the diffusion equation, the smaller diffusion activation energy is, the higher the temperature is, the easier the diffusion is [42]. The process of heat treatment is a process of elements homogenization. During the heat treatment process, the morphology transformation of eutectic silicon phase will go through the following three stages: fracture of eutectic Si phase, roundness and coarsening [43]. During the solidification of the AlSi7Mg0.6 aluminum alloy, the energy state of eutectic silicon phase will be not uniform because the distribution of temperature is unstable. In the process of heat treatment, silicon atoms will migrate from the high energy region to the low energy region, Si phase in higher energy state will slowly dissolve and Si phase in lower energy state will slowly grow up, resulting in the appearance of the rounded Si phase finally [44]. However, Si particles will grow rapidly with increasing holding time during the process of heat treatment. Likewise, the diffusion of elements also makes the distribution of elements more uniform after T6 heat treatment. 

### 4.2. Analysis of the Mechanical Property

Though the average grain size in face XOY and face XOZ of the as-deposited samples is similar, equiaxed grains in face XOY account for a larger proportion than that in face XOZ. That is to say, there are more grain boundaries in face XOY, resulting in a higher elongation in X direction. During the tensile process, there will be stress concentration at the grain boundaries because of elastic deformation and plastic deformation. For one thing, elastic deformation and plastic deformation at the grain boundaries are not in harmony, inducing the stress concentration at the grain boundaries to maintain the continuity of the grains. For another, the existence of grain boundary makes it difficult for the slip dislocation to cross the grain boundary directly, thus damaging the continuity of the slip system and hindering the movement of the dislocation. Equiaxed grains increase grain boundaries and make the dislocation move in more grains, resulting in more uniform plastic deformation and improving the ductility [42]. That is the reason why the elongation in X direction of the as-deposited sample is little higher than that in Z direction.

After T6 heat treatment, the increase of grain size will reduce the strength of the samples. However, the increase of grain size usually accounts for a small proportion in the strength change of the alloy. According to the research results of Callais [45], when the grain size of aluminum alloy is 100 μm, the contribution of intercrystalline strengthening to the enhancement effect is only 2 MPa, while when the grain size is refined to 10 μm, the enhancement effect is only 6.3 MPa. That is to say, the change of yield strength caused by the growth of grain size can be ignored.

After T6 heat treatment, the increase of strength of the samples is caused by precipitation strengthening and the roundness of Si phase. For Al-Si-Mg alloy, the general precipitation sequence is described as: super-saturated solid solution (SSSS) → clusters/GP zones (vacancy-rich zones) → *β″* (coherent precipitates) → *β′* (semi-coherent precipitates) → *β* (incoherent precipitates). The fully coherent monoclinic needle-like *β″* phase is considered as the most effective strengthening precipitate, although there is some discussion about the lattice parameters of *β″* phase [46,47]. The increase of yield strength caused by the precipitated phase could be expressed in the following formula [48]:

(4) $$\Delta \sigma_{y}=\left(0.538Gbf^{1/2}/X\right)\times \ln\left(X/2b\right)$$

where $\Delta \sigma_{y}$ is the increase of yield strength (MPa), *G* is the shear modulus (MPa), *b* is the Burgers vector (nm), *f* is the volume fraction of particles, and *X* is the real diameter of the particles (nm). For aluminium alloy, the value of *G* and *b* is 26.2 GPa and 0.286 nm respectively [49]. In addition, the elasticity modulus of Si phase is very high, reaching 140–150 GPa, so the solid solution strengthening Si is more helpful to the improvement of strength [50]. From another perspective, the morphology of Si phase will have a great influence on the properties of Al-Si alloy.

**Figure 8:**
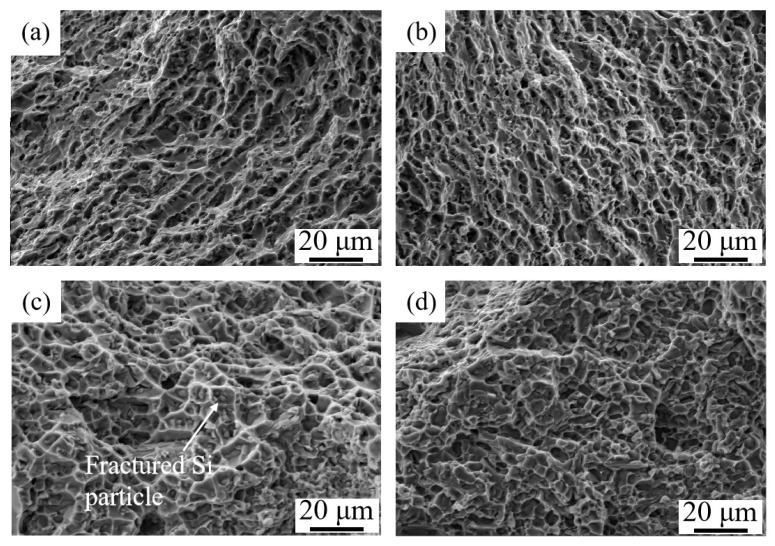
Fracture morphology of tensile tested specimens: (**a**) X direction of the as-deposited samples; (**b**) Z direction of the as-deposited samples; (**c**) X direction of the T6 heat-treated samples; (**d**) Z direction of the T6 heat-treated samples.

After T6 heat treatment, the ductility of samples has no reduction, which is due to the higher work hardening rate caused by nanosized precipitate. The precipitate can storage lots of Orowan dislocation loops and lead to a higher work hardening rate, which thus restrains the necking instability or fracture. The similar researches and toughening mechanism by precipitates have been reported previously. It is proved that proper heat treatment can help the material processed by WAAM-CMT to obtain excellent comprehensive mechanical properties. Fracture morphologies seem to confirm this result, as shown in Figure 8a–d. It can be observed that there is no significant difference among the fracture morphologies in X direction and Z direction of the as-deposited and T6 heat-treated samples. What is more, the dimples dominate the fracture surfaces, reflecting the fact that most of the failure is the result of ductile fracture. Besides, there are fractured Si particles at the bottom of dimples, indicating the fact that the fractured Si can form micro-cracks. 

## 5. Conclusions

With the purpose of improving the rapid cooling microstructure and enhancing the mechanical properties of samples processed by WAAM-CMT, the effects of heat treatment on microstructure and mechanical properties are conducted systematically in the case of AlSi7Mg0.6 thin-wall parts. The following conclusions can be drawn:

1. There are nonuniform grain structure of the as-deposited samples, such as mixture of coarse grain and fine grain zones, blending of columnar and equiaxed grain zones and the segregation of elements, which seems to can be reduced or even eliminated by T6 heat treatment.
2. After T6 heat treatment, the elasticity modulus, the yield strength and the ultimate tensile strength in X and Z directions of the samples all increase obviously, but the elongation of the samples does not have obvious change in two directions.
3. The fractures of the as-deposited and T6 heat-treated samples all are the result of ductile fracture. After T6 heat treatment, the increase of strength of the samples is mainly caused by precipitation strengthening and the roundness of Si phase, and no reduction in ductility is due to the higher work hardening rate caused by nanosized precipitate.

## Figures and Tables

**Table 1:** The chemical composition of AlSi7Mg0.6 wire and samples (mass fraction, wt%).

Element	Si	Mg	Ti	Fe	Al
Wire	6.98	0.66	0.10	0.04	Bal.
Upper location of the sample	6.95	0.65	0.11	0.01	Bal.
Lower location of the sample	6.79	0.63	0.11	0.01	Bal.

**Table 2:** The mechanical properties of the samples before and after heat treatment.

State	m_E_/(GPa)	R_p0.2/(MPa)_	R_m/(MPa)_	A/(%)
As-deposited samples	50.30 ± 3.68 (X)	68.51 ± 2.28 (X)	125.08 ± 8.89 (X)	7.08 ± 0.96 (X)
	54.42 ± 2.83 (Z)	65.64 ± 3.52 (Z)	130.24 ± 9.53 (Z)	6.13 ± 0.72 (Z)
T6 heat-treated samples	69.31 ± 4.11 (X)	255.17 ± 14.42 (X)	351.75 ± 6.77 (X)	6.55 ± 0.70 (X)
	77.26 ± 4.63 (Z)	274.62 ± 14.67 (Z)	353.49 ± 8.16 (Z)	6.20 ± 0.48 (Z)
